# Thoracic aortic microcalcification activity in combined positron emission tomography and magnetic resonance imaging

**DOI:** 10.1007/s00259-024-06670-5

**Published:** 2024-03-08

**Authors:** Jennifer Nash, Samuel Debono, Beth Whittington, Jakub Kaczynski, Tim Clark, Gillian Macnaught, Scott Semple, Edwin J R van Beek, Adriana Tavares, Damini Dey, Michelle C Williams, Piotr J Slomka, David E Newby, Marc R Dweck, Alexander J Fletcher

**Affiliations:** 1 The University of Edinburgh Centre for Cardiovascular Science, University of Edinburgh, Room SU.305, Chancellor’s Building, 49 Little France Crescent, Edinburgh, EH16 4SB UK; 2 Department of Medical Physics, NHS Lothian, Royal Infirmary of Edinburgh, Edinburgh, UK; 3 Edinburgh Imaging Facility Queens Medical Research Institute, University of Edinburgh, Edinburgh, UK; 4 Departments of Medicine, Division of Artificial Intelligence) and Biomedical Imaging Research Institute, Cedars-Sinai Medical Centre, Los Angeles, USA; 5 School of Cardiovascular and Metabolic Health, University of Glasgow, Glasgow, UK

**Keywords:** Positron emission tomography and magnetic resonance imaging, Sodium [^18^F]fluoride, Thoracic aorta, Attenuation correction

## Abstract

**Introduction:**

Non-invasive detection of pathological changes in thoracic aortic disease remains an unmet clinical need particularly for patients with congenital heart disease. Positron emission tomography combined with magnetic resonance imaging (PET-MRI) could provide a valuable low-radiation method of aortic surveillance in high-risk groups. Quantification of aortic microcalcification activity using sodium [^18^F]fluoride holds promise in the assessment of thoracic aortopathies. We sought to evaluate aortic sodium [^18^F]fluoride uptake in PET-MRI using three methods of attenuation correction compared to positron emission tomography computed tomography (PET-CT) in patients with bicuspid aortic valve,

**Methods:**

Thirty asymptomatic patients under surveillance for bicuspid aortic valve disease underwent sodium [^18^F]fluoride PET-CT and PET-MRI of the ascending thoracic aorta during a single visit. PET-MRI data were reconstructed using three iterations of attenuation correction (Dixon, radial gradient recalled echo with two [RadialVIBE-2] or four [RadialVIBE-4] tissue segmentation). Images were qualitatively and quantitatively analysed for aortic sodium [^18^F]fluoride uptake on PET-CT and PET-MRI.

**Results:**

Aortic sodium [^18^F]fluoride uptake on PET-MRI was visually comparable with PET-CT using each reconstruction and total aortic standardised uptake values on PET-CT strongly correlated with each PET-MRI attenuation correction method (Dixon *R* = 0.70; RadialVIBE-2 *R* = 0.63; RadialVIBE-4 *R* = 0.64; *p* < 0.001 for all). Breathing related artefact between soft tissue and lung were detected using Dixon and RadialVIBE-4 but not RadialVIBE-2 reconstructions, with the presence of this artefact adjacent to the atria leading to variations in blood pool activity estimates. Consequently, quantitative agreements between radiotracer activity on PET-CT and PET-MRI were most consistent with RadialVIBE-2.

**Conclusion:**

Ascending aortic microcalcification analysis in PET-MRI is feasible with comparable findings to PET-CT. RadialVIBE-2 tissue attenuation correction correlates best with the reference standard of PET-CT and is less susceptible to artefact. There remain challenges in segmenting tissue types in PET-MRI reconstructions, and improved attenuation correction methods are required.

**Supplementary Information:**

The online version contains supplementary material available at 10.1007/s00259-024-06670-5.

## Introduction

Positron emission tomography with magnetic resonance imaging (PET-MRI) is a modality with an evolving utility in cardiovascular disease, offering simultaneous acquisition of functional, anatomical and molecular information. It has been demonstrated to be robust and feasible in cardiac conditions [1], but its applicability in aortic disease has yet to be established.

Bicuspid aortic valve disease is associated with aortopathy where there is an abnormal dilatation of the ascending aorta and risk of secondary aortic dissection [2]. Acute aortic dissection is commonly the first presentation of symptomatic aortic disease and is usually fatal [3]. Standard clinical care relies on surveillance of the aortic diameter, with targeted prophylactic intervention when the aorta reaches a threshold diameter to trigger surgical intervention. Despite this, a high proportion of patients with aortopathy experience aortic dissection when the thoracic aortic diameter is below the threshold for surgical intervention [2]. There is a clear need to improve clinical risk stratification in patients with bicuspid aortic valve associated aortopathy.

The characterisation of early pathophysiological change in the aortic wall is an area of major interest in bicuspid aortic valve related aortopathy. Microcalcification occurs because of breaks in elastin fibre complexes within the media of the aorta, resulting in the deposition of microscopic hydroxyapatite crystals [4, 5]. Sodium [^18^F]fluoride is a radiotracer which binds to hydroxyapatite crystals and can be detected and quantified using PET [6, 7]. Studies using sodium [^18^F]fluoride PET combined with computed tomography (CT) have shown promise in patients with aortic dissections. In a cohort of patients suffering acute aortic dissection, sodium [^18^F]fluoride uptake at the dissection site was associated with aortic growth and major adverse aortic events [8].

PET-MRI poses several potential advantages over PET-CT as it confers lower ionising radiation exposure, excellent soft tissue contrast, and dynamic functional assessment of blood flow [9]. These benefits make it a potentially attractive modality for monitoring disease activity in thoracic aortopathies. However, an ongoing challenge in PET-MRI is attenuation correction during data processing. A common approach is to generate an attenuation correction map using the Dixon MRI sequence [10], but this technique is sensitive to motion and breathing related artefact particularly at the borders between the lung and both the heart and the diaphragm [11, 12]. An alternative technique is utilising free-breathing radial gradient recalled echo (GRE) sequences which can reduce this artefact [11].

In this study, we aimed to evaluate the assessment of thoracic aortic sodium [^18^F]fluoride uptake on PET-MRI in adults with bicuspid aortic valve disease. We sought to compare three separate PET-MRI attenuation correction methods against the reference standard of PET-CT.

## Materials and methods

### Study population

Thirty subjects underwent sodium [^18^F]fluoride PET-CT and PET-MRI examinations as part of the Assessment of Risk in Thoracic Aortopathy using sodium [^18^F]fluoride study (AoRTAs; NCT04083118). All participants were under surveillance for bicuspid aortic valve disease (diagnosed with transthoracic echocardiography with or without adjunct CT or MRI) and were aged over 40 years. Subjects with previous aortic root replacement, previous aortic dissection or rupture, contrast allergy or pregnancy were excluded. The study was approved by the Scottish Research Ethics Committee (REC reference: 18/SS0136), the United Kingdom Administration of Radiation Substances Advisory Committee and local institutional review board. Written informed consent was obtained from all participants.

### PET-CT acquisition

All participants were administered a target dose of 250 MBq sodium [^18^F]fluoride intravenously and were imaged after 60 min using a hybrid 128-slice PET-CT scanner (Biograph mCT, Siemens Healthineers, Germany). A low-dose attenuation correction CT scan was performed (100–120 kV, 40–50 mAs, 5/3 mm). PET data were acquired using electrocardiogram (ECG)-gating in list-mode in three 10-min bed positions ensuring coverage of the entire thoracic aorta and heart. PET-CT images were corrected for attenuation, dead time, scatter and random coincidences, using an optimised iterative reconstruction algorithm (ultra-HD; TrueX + Time of Flight, matrix 200, zoom 1; 5 mm Gaussian filter).

### PET-MRI acquisition

Upon completion of the PET-CT examination, the subjects immediately underwent PET-MRI on a hybrid PET-MRI scanner (Biograph mMR, Siemens Healthineers, Germany) allowing simultaneous MRI and PET data acquisition. Using PET compatible elements from a 12-channel body matrix and a spine matrix coils, all images were acquired using ECG-gating. Both Dixon (end-expiration, breath-held, 3-dimensional, dual-echo spoiled gradient-recalled echo [10]) and free-breathing radial GRE (gradient recalled echo; RadialVIBE [11, 12]) sequences were acquired. Following acquisition, PET data were reconstructed using the following parameters to provide optimum signal-to-noise and contrast-to-noise ratios: 344 matrix, 3 iterations, 21 subsets, 5-mm Gaussian filter. Three final attenuation correction maps were generated based upon MRI sequences performed: a standard Dixon map (segmentation into 4 tissue types: air, lung, soft tissue and fat) and two custom attenuation correction maps based on radial GRE (RadialVIBE-2 with segmentation into 2-tissue types [lung/air and soft tissue]) and RadialVIBE-4 with segmentation into 4-tissue types [soft tissue, lung, fat, background air]). All analyses were performed on the ordered subsets expectation maximisation (OSEM) algorithm image reconstructions.

### Aortic valve assessment

Aortic valve morphology was assessed using aortic valve CINE images and classified using international consensus classification [13]. The aortic valve function was assessed using 2-dimensional phase contrast magnetic resonance images at the sinotubular junction (with the velocity encoded threshold set to the lowest value without aliasing). Aortic valve velocities and velocity gradients were assessed by transthoracic echocardiography. Valve stenosis was determined using the maximum aortic velocity and graded as none (< 2.0 m/s), mild (2.0-2.9 m/s), moderate (3.0-3.9 m/s) or severe (≥ 4.0 m/s) [14]. Valve regurgitation was assessed using cardiac MRI images and graded as none, mild, moderate or severe based on visual assessment of the valve, the presence or absence of flow reversal in the thoracic aorta and regurgitation fraction by an accredited consultant with specialist expertise in MRI blinded to the PET results (MRD) (calculated using 2-dimensional phase contrast sequences and stroke volume differential).

### Analysis of Aortic Sodium [^18^F]Fluoride Uptake

PET-MRI images were qualitatively assessed visually for similarity to PET-CT images and the presence of artefact at tissue type interfaces by one investigator (JN). Quantitative sodium [^18^F]fluoride uptake was assessed using FusionQuant v1.21.0421 software (Cedars-Sinai Medical Centre, Los Angeles). Images from PET-CT and each PET-MRI reconstruction were co-registered in three orthogonal planes using magnetic resonance angiography. Background blood pool activity was measured by drawing one 2.1-cm^3^ sphere of interest in both the right and left atria and calculating the average standardised uptake value divided by volume (background SUVmean).

**Fig. 1:**
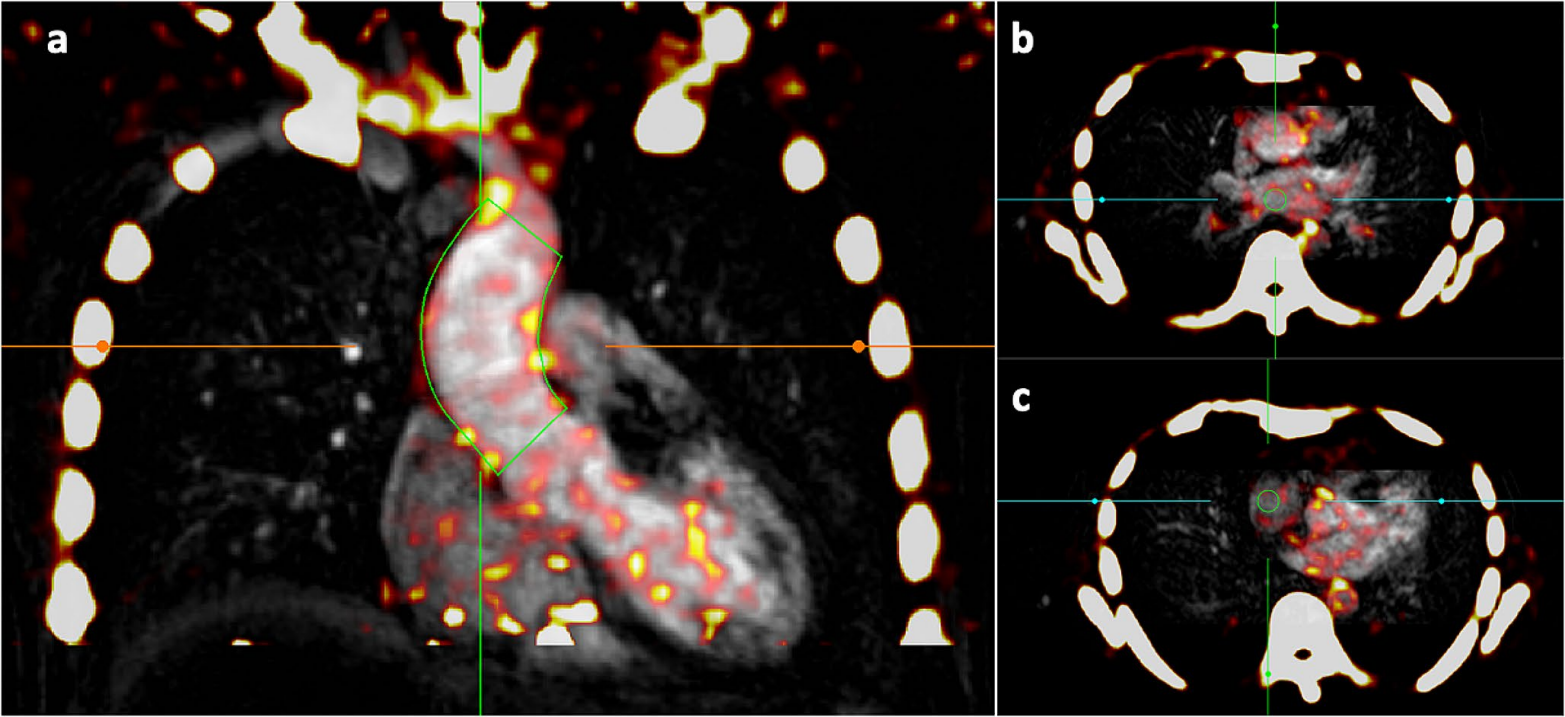
Method of determining aortic sodium [^18^F]fluoride activity in positron emission tomography (PET) images. PET images are first co-registered with magnetic resonance angiography in three orthogonal planes. (**a**) The ascending aortic activity is measured by drawing a centreline from the sinotubular junction to the brachiocephalic artery (green line). Calculated values are total standardised uptake value (total aortic SUV), mean standardised uptake value (aortic SUVmean), maximum standardised uptake value (SUVmax) and aortic microcalcification activity (AMA). Blood pool estimates are obtained by drawing a 2.1-cm^3^ sphere of interest (green circle) in the left atrium (**b**) and right atrium (**c**)

Previously described method of determining the total standardised uptake value (total SUV), average aortic standardised uptake value (SUVmean) and the maximum standardised uptake value (SUVmax) were used for assessment of the ascending aorta. This method has been shown to be highly reproducible and repeatable in aortic PET quantification [15, 16] (Fig. 1).

In brief, the images were analysed using a centreline-based approach to create a volume of interest. Starting at the sinotubular junction and ending at the brachiocephalic artery, a centreline was created at a minimum of three levels within the ascending aorta using a multiplanar reconstruction viewer. The diameter was drawn to the maximal internal aortic diameter then increased by 4 mm. Contouring around the aorta was drawn using a variable radius function to accommodate any anatomical vessel tortuosity or focal dilatation and reduce partial volume effects. The variable radius function has been demonstrated to improve sodium [^18^F]fluoride quantification in abdominal aortic aneurysm analysis as described previously [16]. After drawing the target volume of interest, total SUV, SUVmean (total SUV within the aorta divided by its volume) and SUVmax (maximum voxel intensity with the drawn region of interest) were generated. Aortic microcalcification activity (AMA) was calculated as the cumulative voxel intensity in the region of interest divided by its volume and corrected for background sodium [^18^F]fluoride activity as described previously [16].

### Statistical analysis

Statistical analysis was performed using the software package R (v4.0.2, R Foundation Statistical Computing, Vienna). Shapiro-Wilk tests were used to assess normality of distribution of results. Categorical variables were presented as number (percentage). Continuous variables with normal distribution were present as mean (± standard deviation), and non-normally distributed variables were presented as median [interquartile range]. Associations between sodium [^18^F]fluoride uptake in PET-CT and PET-MRI and associations between aortic valve mean pressure gradient and sodium [^18^F]fluoride uptake were evaluated as a continuous variable (Spearman’s correlation coefficient). Correlation coefficients (R) of 0-0.19 were regarded as very weak, 0.2–0.39 as weak, 0.40–0.59 as moderate, 0.6–0.79 as strong and 0.8-1 as very strong. Agreements between sodium [^18^F]fluoride uptake in PET-CT and each PET-MRI reconstruction were assessed using mean bias, 95% limits of agreement, intraclass correlation coefficient (consistency and two-way random effects model) [17] and Bland-Altman plots [18]. Intraclass correlation coefficient values was described as poor when less than 0.5, moderate when 0.5–0.75, good when 0.75–0.9 and excellent when greater than 0.9 [17]. Mean sodium [^18^F]fluoride uptake values as quantified by AMA, total aortic SUV, aortic SUVmean, aortic SUVmax and blood pool were compared using a one-way ANOVA and post-hoc paired t-tests. Statistical significance was set as a two-sided *p* < 0.05.

## Results

The study population consisted of 30 consecutive patients with bicuspid aortic valve with a mean age of 49 years, 27% (9/30) were female and the average ascending aortic diameter was 41 mm (Table 1, Supplementary Table 1). Most participants had mild or no valve stenosis (*n* = 26, 87%) or regurgitation (*n* = 25, 83%). There was no demonstrable relationship between bicuspid aortic valve mean pressure gradient and aortic sodium [^18^F]fluoride uptake in any reconstruction or quantification method (Supplementary Fig. 1, Supplementary Fig. 2).

**Table 1:** Patient characteristics

Characteristic	*N* = 30
Age (years)	48.7 ± 5.1
Female sex	9 (27)
Body mass index (kg/m^2^)	28.4 ± 4.8
Body surface area (m^2^)	2.04 ± 0.2
Systolic blood pressure (mmHg)	132 ± 15.9
Diastolic blood pressure (mmHg)	79 ± 9
Heart rate (beats/min)	63 ± 12
*Medical history*	
Bicuspid aortic valve	30 (100)
History of cerebrovascular disease	1 (3.3)
History of ischaemic heart disease	0 (0)
History of hypertension	9 (30)
History of diabetes mellitus	0 (0)
History of coarctation of the aorta	3 (10)
*Smoking status*	
Smoker	4 (13)
Ex-smoker	4 (13)
Never smoked	22 (73)
*Medication*	
Angiotensin-converting enzyme inhibitor or angiotensin II receptor blocker	12 (40)
Beta-blocker	3 (10)
Statin	0 (0)
Antiplatelet	2 (7)
*Aortic valve*	
Bicuspid aortic valve subtype	
Fused	
*Right-left cusp fusion*	19 (63)
*Right-non cusp fusion*	5 (17)
*Left-non cusp fusion*	0 (0)
*Indeterminant cusp fusion*	0 (0)
2-sinus	
*Latero-lateral*	3 (10)
*Anterior-posterior*	3 (10)
Partial-fusion	0 (0)
Aortic stenosis	
* None*	17 (57)
*Mild*	9 (30)
*Moderate*	4 (13)
*Severe*	0 (0)
Aortic regurgitation	
*None*	20 (67)
*Mild*	5 (17)
*Moderate*	4 (13)
*Severe*	1 (3)
*Aorta*	
Aortic root diameter (mm)	38.2 [6.6]
Ascending aorta diameter (mm)	40.50 ± 4.46
Aortic size index (root) (cm/m^2^)	19.5 ± 2.62
Aortic size index (ascending) (cm/m^2^)	20.05 ± 2.79

Mean ± standard deviation; number (%), median [interquartile range]

### Image Quality

**Fig. 2:**
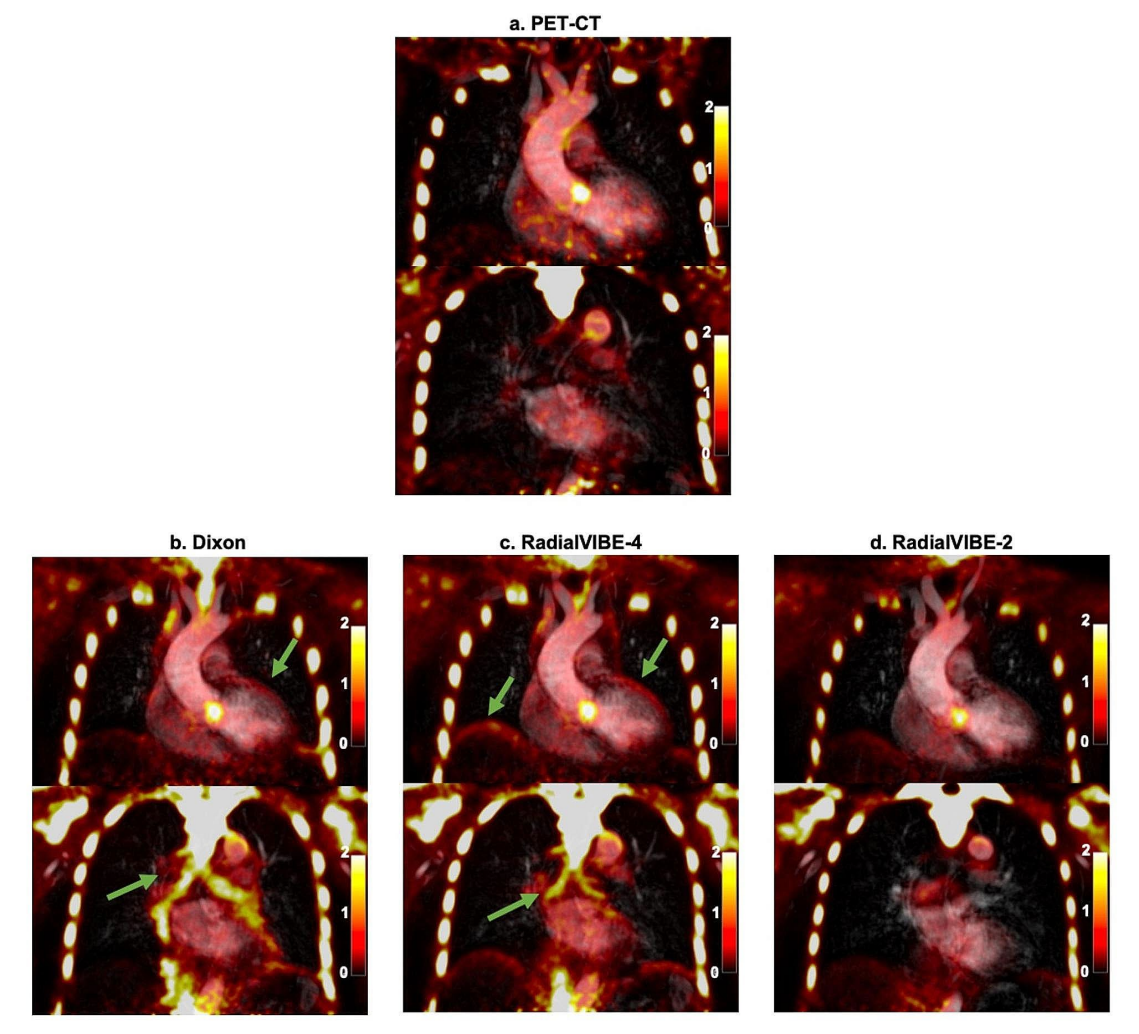
Thoracic sodium [^18^F]fluoride uptake in two coronal planes in a patient with bicuspid aortic valve. (**a**) Positron emission tomography (PET)-computed tomography (CT) co-registered with magnetic resonance angiography (MRA), (**b**) combined Dixon attenuation correction PET and MRA, (**c**) combined RadialVIBE-4 attenuation correction PET and MRA, and (**d**) combined RadialVIBE-2 attenuation correction PET and MRA. Sodium [^18^F]fluoride uptake in each PET-MRI image demonstrates a similar pattern compared with uptake in PET-CT. Note the image artefact in Dixon and RadialVIBE-4 at the bronchial tree and heart-lung and diaphragm-lung border (green arrows). Colour scale on the right of images represents standardised uptake values

Patterns and distribution of radiotracer activity were similar per patient in each PET reconstruction. As previously identified, Dixon reconstructions were consistently affected by increased radiotracer activity at the heart-lung border, lung-diaphragm border and within the bronchi in all participants. Artefact in these regions was also observed in RadialVIBE-4 reconstructions but were not present in RadialVIBE-2 images. No tissue interfacing artefact was detected in any PET-CT image (Fig. 2).

### Image Quantification

**Fig. 3:**
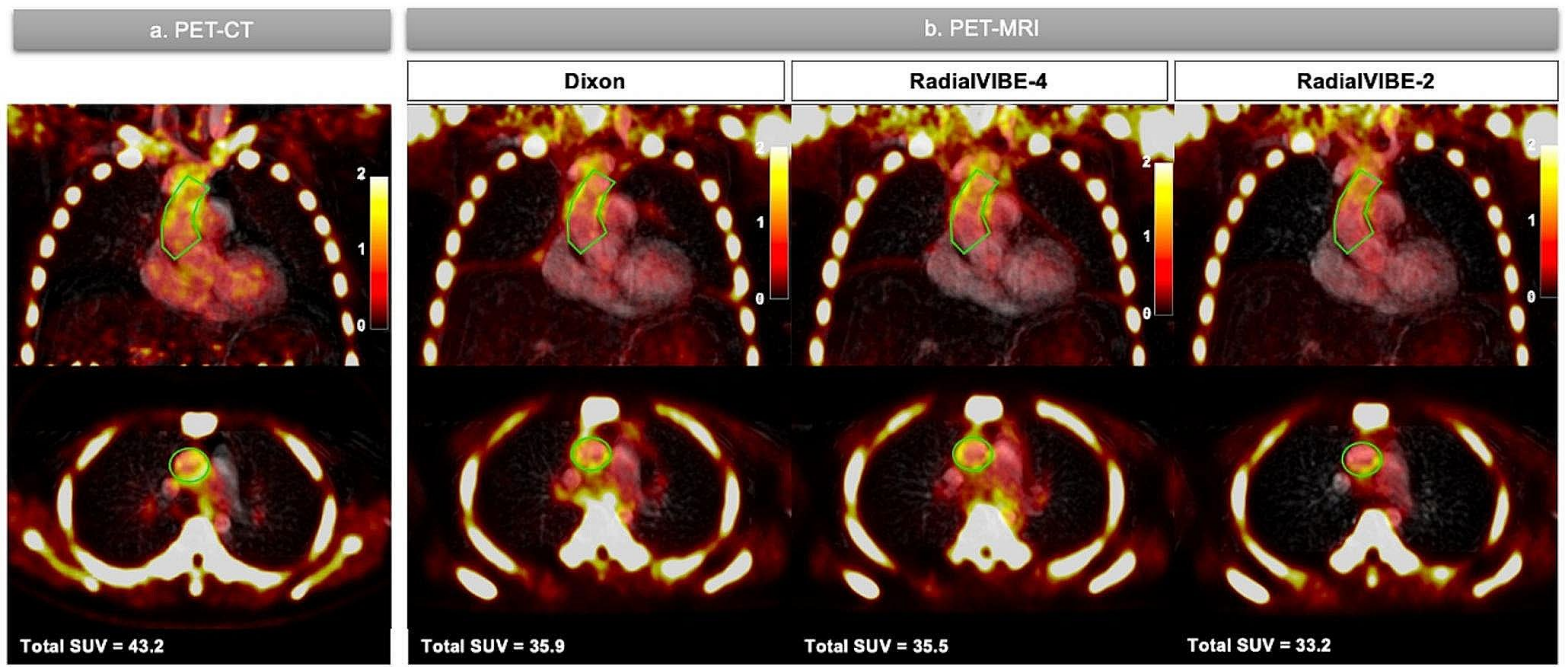
Representative coronal and axial images of ascending aortic sodium [^18^F]fluoride quantification in positron emission tomography (PET) images of a patient with bicuspid aortic valve. (**a**) PET-computed tomography (CT) co-registered with magnetic resonance angiography (MRA). (**b**) PET-magnetic resonance imaging (MRI) combined with MRA in each attenuation correction method (Dixon, RadialVIBE-4 and RadialVIBE-2). Green line represents region of interest around the ascending aorta. Colour scale on the right of images represents standardised uptake values. Total SUV = total standardised uptake value

Sodium [^18^F]fluoride uptake was detected in the aorta of all subjects, although intensity and distribution were variable across individual patients (Table 2; Fig. 3). Total aortic SUV ranged from 40.1 to 159.8 in PET-CT images, 35.9 to 143.2 in Dixon images, 35.5 to 139.1 in RadialVIBE-4 images and 31.4 to 146.5 in RadialVIBE-2 images. Aortic SUVmax ranged from 0.95 to 3.66 in PET-CT, 1.71 to 6.10 in Dixon, 1.34 to 4.44 in RadialVIBE-4 and 1.02 to 3.32 in RadialVIBE-2. There was no demonstrable difference in total aortic SUV, aortic SUVmean or AMA values in each PET-MRI reconstruction compared to PET-CT values of each measure (Table 2). Aortic SUVmax measurements were higher in Dixon and RadialVIBE-4 reconstructions compared to PET-CT. Lower blood pool activity estimates were observed in Dixon and RadialVIBE-4 than PET-CT.

**Table 2:** Sodium [^18^F]fluoride uptake and activity in the aorta and blood pool in each positron emission tomography reconstruction

PET reconstruction	Total aortic SUV	Aortic SUVmean	Aortic SUVmax	AMA	Blood pool
PET-CT	82.7 ± 27.5	0.92 ± 0.19	1.98 ± 0.68	1.25 ± 0.17	0.75 ± 0.16
Dixon PET-MRI	77.1 ± 25.1	0.91 ± 0.17	2.54 ± 0.87**	1.35 ± 0.25	0.69 ± 0.12*
RadialVIBE-4 PET-MRI	79.4 ± 26.8	0.95 ± 0.19	2.25 ± 0.77***	1.27 ± 0.03	0.75 ± 0.14
RadialVIBE-2 PET-MRI	75.2 ± 26.1	0.88 ± 0.16	1.86 ± 0.66	1.28 ± 0.18	0.69 ± 0.11*

Aortic microcalcification activity (AMA) is calculated by dividing the total aortic SUV by its volume, then dividing by the mean blood pool

Results are presented as mean ± standard deviation. There was no difference in average total aortic SUV, aortic SUV or AMA amongst modalities. RadialVIBE-4 had higher average blood pool activity than Dixon and RadialVIBE-2. Aortic SUVmax was significantly higher in Dixon and RadialVIBE-4 compared to RadialVIBE-2.

Statistical comparisons highlighted are of differences observed in SUVmax (compared to RadialVIBE-2) and blood pool (compared to RadialVIBE-4). *p < 0.05, **p < 0.01, ***p < 0.001

PET-CT = positron emission tomography – computed tomography, PET-MR = positron emission tomography – magnetic resonance, SUVmax = maximum standardised uptake value, SUVmean = mean standardised uptake value, AMA = aortic microcalcification activity, total aortic SUV = total aortic standardised uptake value

Strong correlations in total aortic SUV were detected between PET-CT and each PET-MRI reconstruction (PET-CT versus Dixon, *R* = 0.70; PET-CT versus RadialVIBE-4, *R* = 0.64; PET-CT versus RadialVIBE-2, *R* = 0.63; *p* < 0.001 for all). After adjusting for the volume in each region of interest, PET-CT values remained strongly correlated with Dixon (*R* = 0.62, *p* < 0.001) and RadialVIBE-2 (*R* = 0.60, *p* < 0.001), with a moderate correlation observed with RadialVIBE-4 (*R* = 0.47, *p* = 0.01). When correcting for blood pool using AMA, there were moderate correlations between PET-CT and Dixon (*R* = 0.46, *p* = 0.01) or RadialVIBE-2 (*R* = 0.47, *p* = 0.009) but no demonstrable correlation between PET-CT and RadialVIBE-4 (*R* = 0.29, *p* = 0.13). Aortic SUVmax was moderately correlated between PET-CT and RadialVIBE-2 (*R* = 0.51, *p* = 0.004), but there was no observed correlation between PET-CT and Dixon (*R*=-0.15, *p* = 0.44) or PET-CT and RadialVIBE-4 (*R* = 0.17, *p* = 0.37).

**Fig. 4:**
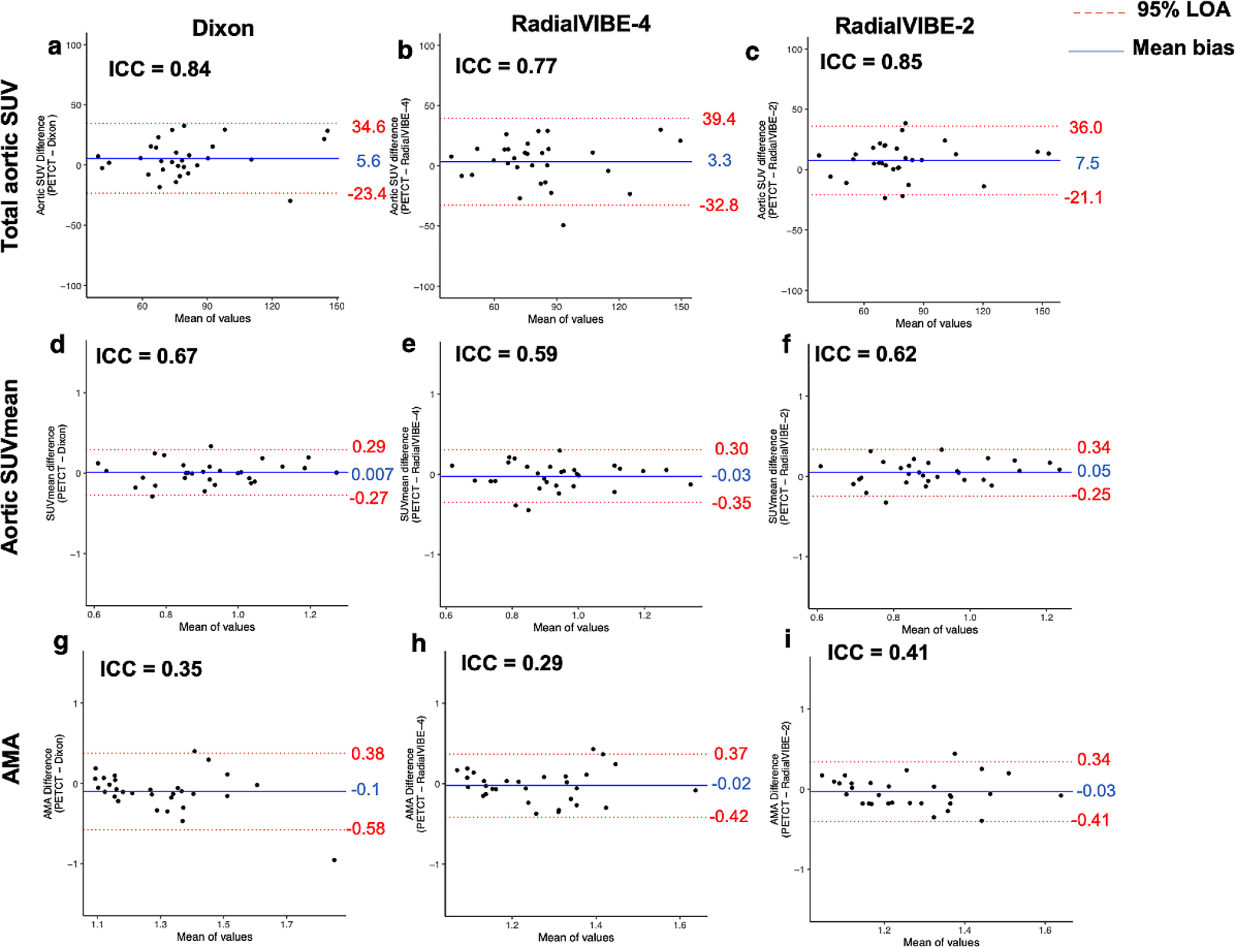
Levels of agreement are assessed in the three different methods of measuring aortic sodium [^18^F]fluoride uptake. Bland-Altman plots demonstrate percentage difference in values with mean bias (blue line) and 95% limits of agreement (red lines) for aortic sodium [^18^F]fluoride activity. The columns represent the PET (positron emission tomography) and magnetic resonance imaging (MRI) attenuation correction method compared with PET and computed tomography (CT) method. (**a**-**c**) represent total aortic standardised uptake value (SUV), (**d**-**f**) represent aortic mean standardised uptake (SUVmean), and (g-i) represent aortic microcalcification activity (AMA). ICC = intraclass correlation coefficient, LOA = limits of agreement

**Fig. 5:**
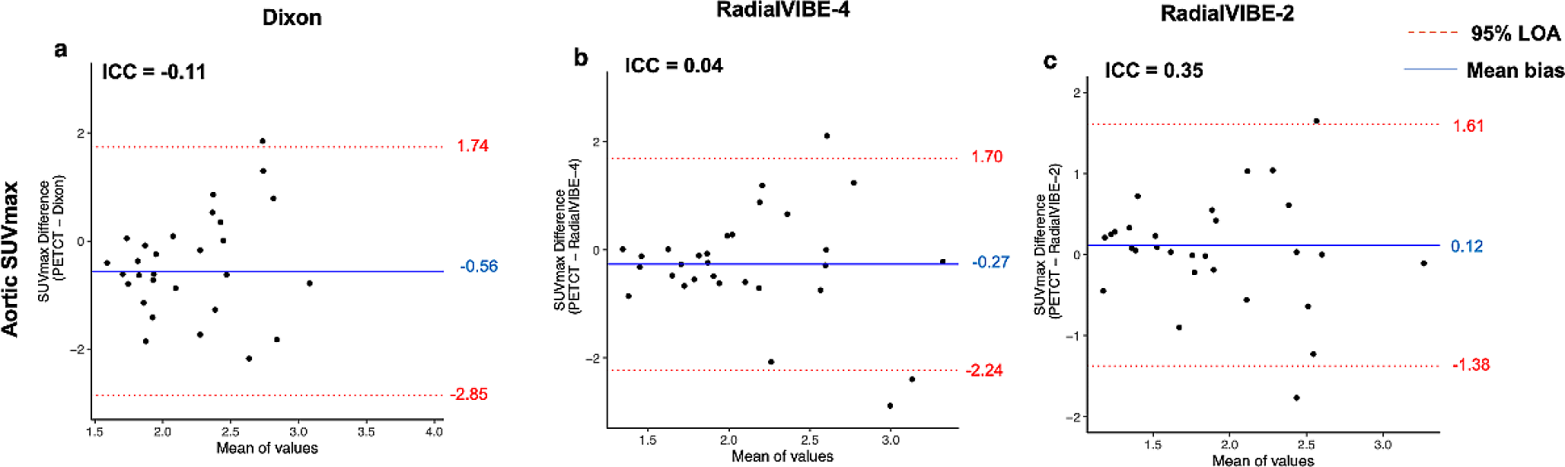
Levels of agreement in maximum standardised uptake value (SUVmax) of aortic sodium [^18^F]fluoride uptake. Bland-Altman plots demonstrates percentage difference in values with mean bias (blue line) and 95% limits of agreement (red lines) for maximum aortic sodium [^18^F]fluoride activity. (**a**) Dixon positron emission tomography (PET) - magnetic resonance imaging (MRI) attenuation correction method compared with PET-computed tomography (CT) method, (**b**) RadialVIBE-4 compared with PET-CT, and (**c**) RadialVIBE-2 compared with PET-CT. ICC = intraclass correlation coefficient, LOA = limits of agreement, SUVmax = maximum standardised uptake value

Mean bias between each PET-MRI reconstruction and PET-CT were generally consistent across each quantification method (ranging from 14.6 to 18.0% in total SUV, 12.3–14.5% in SUVmean, 12.3–13.4% in AMA; Fig. 4). SUVmax measurements yielded higher mean bias between each PET-MRI reconstruction and PET-CT than SUVmean based calculations (25.9–39.6%; Fig. 5). Limits of agreement were similarly found to be consistent in total SUV and SUVmean, with narrower limits of agreement observed in RadialVIBE-2 in SUVmax and AMA calculations (Figs. 4 and 5).

RadialVIBE-2 demonstrated the strongest and most consistent agreement with PET-CT in AMA, aortic SUVmean, total aortic SUV and aortic SUVmax in comparison with the other PET-MRI reconstructions (Figs. 4 and 5, Supplementary Table 2, Supplementary Fig. 1, Supplementary Fig. 2). In SUVmean based activity measurements, weaker limits of agreement were driven by variability observed in blood pool activity across PET-MRI reconstructions. RadialVIBE-4 mean blood pool activity (0.75 ± 0.14) was higher than both Dixon (0.69 ± 0.12; *p* = 0.02) and RadialVIBE-2 (0.69 ± 0.11, *p* = 0.02; Table 2).

## Discussion

In this study, we have demonstrated that sodium [^18^F]fluoride aortic quantification using PET-MRI is feasible, with comparable results to PET-CT and with the mean aortic standardised uptake values correlating strongly with each PET-MRI attenuation correction method. However, there are important differences between the three methods of PET-MRI attenuation correction and the reference standard of PET-CT. We have found that a radial GRE technique with two-tissue segmentation performs most consistently compared with four-tissue or Dixon attenuation correction methods.

Attenuation correction in PET-MRI is a considerable challenge, and there remains a lack of consensus in the optimal method to provide accurate attenuation [19]. Photon attenuation can result in up to 90% signal reduction [20], leading to potential errors in PET quantification. Indeed, there is risk of substantial underestimation of PET signal if inadequate attenuation correction is applied when processing sodium [^18^F]fluoride PET images, especially given the ascending aorta lies beneath the sternum. Improving PET-MRI attenuation maps that are more aligned with PET-CT is necessary to ensure it can reach the robustness of this widely employed modality. Here, we have demonstrated that PET-MRI attenuation correction can generate images and standardised uptake values that are comparable to PET-CT when examining the thoracic aorta.

Prior work has favoured radial GRE over Dixon as comparators to PET-CT when quantifying sodium [^18^F]fluoride activity in aortic valve disease [11]. In theory, further tissue separation could facilitate more specific discrimination in tissue uptake and improve agreements to PET-CT. However, we have found that the four-tissue radial GRE does not improve sodium [^18^F]fluoride quantification in the aorta. Both Dixon and RadialVIBE-4 are affected by extra-cardiac artefact which alters their qualitative agreement with PET-CT images and increases the risk of overspill in regions of interest. These results would therefore support the use of a two-tissue radial GRE technique for attenuation correction in PET-MRI in thoracic aortic disease.

In this study, we observed mean biases between 12 and 18% in SUVmean based measurements and over 25% in SUVmax measurements compared to PET-CT. Furthermore, we found limits of agreement to be in the order of between 30 and 49% in each SUVmean based reconstruction. In spite of what appear to be relatively wide limits of agreement, we found these limits to be consistent across each PET-MRI reconstruction, with strongest agreements observed in RadialVIBE-2 attenuation corrected images. Future work evaluating the diagnostic and prognostic ability of PET-MRI in this patient group will allow us to determine whether these levels of agreement are clinically acceptable. Moreover, we found no association between aortic valve mean pressure gradient and sodium [^18^F]fluoride uptake in the ascending aorta. These results are consistent with previous studies which found severity of bicuspid aortic valve stenosis was not associated with aortic dilatation status [21]. Prospective study of this patient group is warranted to evaluate the clinical importance of PET-MRI detected ascending aortic microcalcification and its association with disease progression in patients with bicuspid aortic valve.

Perhaps unexpectedly, increasing the number of tissue classes in radial GRE did not improve the agreement with PET-CT. There are several reasons why this could be the case. First, increasing tissue types in radial GRE was associated with artefact detected at the heart-lung border, lung-diaphragm border and the bronchial tree; a similar pattern seen in Dixon reconstructions. Quantification of sodium [^18^F]fluoride activity in the aortic tissue itself was affected by overspill from the bronchus given its close proximity to the ascending portion of the aorta. Given no uptake in the bronchi was detected in PET-CT, the aortic SUVmean measured in RadialVIBE-4 was considerably less reliable than a two-tissue segmentation reconstruction. The artefact zones observed at the heart-lung or lung-diaphragm borders were less problematic when the total aortic SUV was measured in isolation given these artefacts were remote from the aortic region of interest.

Aortic SUVmax in RadialVIBE-2 was the only PET-MRI reconstruction found to be correlated with PET-CT, with no discernible relationship with Dixon or RadialVIBE-4 using this measure. Visually, the high artefactual sodium [^18^F]fluoride uptake in the bronchial tree appeared to contribute substantially to the overspill of signal into the ascending aorta in both Dixon and RadialVIBE-4 attenuation correction methods. Given SUVmax may provide a valuable marker of nonuniform or focal disease activity, such artefact has considerable impact on the reliability of quantitative analysis.

Variability in blood pool measures was a strong determinant of the lack of agreement between PET-CT and both Dixon and RadialVIBE-4 reconstructions. In this study, the blood pool was measured using an average of the uptake in two regions of interest in the left and right atria. This has been shown to be reliable when utilising AMA in PET-CT in the thoracic and abdominal aorta [15, 16]. There is minimal microcalcification activity in the atria and spares the risk of overspill from adjacent bony structures (namely the vertebra) that can be observed when using the superior vena cava. Increased artefactual sodium [^18^F]fluoride uptake as seen in regions of air-soft tissue interface are likely to have affected blood pool quantification in reconstructions where this artefact is apparent.

We should consider the clinical relevance of our work. The utility of imaging biomarkers for risk stratification in thoracic aortic disease is of increasing interest to the international cardiovascular community, and as such, PET imaging techniques are of substantial scientific and clinical importance. In patients with atherosclerotic cardiovascular disease, we have recently demonstrated that thoracic aortic microcalcification (quantified in sodium [^18^F]fluoride PET-CT) is associated with atherosclerotic progression and future ischaemic stroke [22]. Moreover, using sodium [^18^F]fluoride PET-CT, aortic microcalcification is associated with aortic expansion and major adverse aortic events in patients with acute aortic syndrome [8]. Translating molecular imaging techniques to asymptomatic patients under aortic surveillance could provide a valuable mechanism to improve detection of clinical risk. The current standard of monitoring aortic diameter does not adequately capture most patients with thoracic aortopathy who go on to experience major adverse aortic events. In these populations, over 50% of patients who suffer acute aortic dissection do so below the aortic diameter threshold that would trigger prophylactic surgical intervention [2]. Microcalcification resulting from elastin fibre destruction in the aortic wall media could prove a promising target for tracking disease progression. In ex vivo studies, microcalcification in aortic tissue has been demonstrated to be associated with disease severity in aortic aneurysm disease, including in inherited conditions such as Marfan syndrome [4, 23, 24].

In vivo characterisation of microcalcification in aortopathy using PET-MRI poses several advantages over PET-CT. PET-MRI is associated with a substantially reduced radiation exposure and superior soft tissue contrast with the capability of dynamic functional assessments, and so could be a superior imaging strategy in inherited thoracic aortopathy where younger patients require serial surveillance examinations and monitoring of concurrent cardiac pathology. It is therefore of paramount importance to determine if PET-MRI results are comparable to PET-CT. Throughout our analysis, we observed similar patterns of sodium [^18^F]fluoride uptake in each paired PET-CT and PET-MRI scans. This would suggest that PET-MRI could be reassuringly employed in a surveillance protocol of at-risk patients in place of PET-CT, thereby considerably reducing lifetime cumulative radiation dose.

This is the first study to investigate sodium [^18^F]fluoride PET-MRI attenuation correction methods compared to PET-CT in the thoracic aorta. It is important to acknowledge its limitations. This is a small study and validation of our findings in a larger cohort study is warranted. We have included one disease group of bicuspid aortic valve, and we would welcome comparisons in healthy populations and other aortic disease conditions. As alluded to, comparison between PET-CT and PET-MRI is imperfect. Utilising the CT attenuation map to correct PET-MRI could reduce inbuilt errors of comparison, but this technique requires validation. Additionally, there is emerging interest in machine learning methods in PET-MRI attenuation correction, and as such pseudo-CT attenuation correction map generated from MRI has the potential to optimise accuracy of PET-MRI quantification [25].

## Conclusion

Aortic sodium [^18^F]fluoride uptake can be feasibly quantified with PET-MRI, with similar results to conventional PET-CT. A two-tissue radial GRE technique generates findings that are most consistent with PET-CT and is not improved by increasing tissue type segmentation. There are continued issues with air and soft tissue interface artefact in Dixon and four-tissue radial GRE attenuation correction methods, which substantially hampers reliable blood pool activity and maximum standardised uptake value measurement. Further techniques to optimise PET-MRI attenuation correction are warranted.

### Electronic supplementary material

Below is the link to the electronic supplementary material.

Supplementary Material 1

## Data Availability

The datasets generated during and/or analysed during the current study are available from the corresponding author on reasonable request.

## References

[1] Nensa F, Bamberg F, Rischpler C, Menezes L, Poeppel TD, la Fougère C (2018). Hybrid cardiac imaging using PET/MRI: a joint position statement by the European Society of Cardiovascular Radiology (ESCR) and the European Association of Nuclear Medicine (EANM). Eur Radiol.

[2] Fletcher AJ, Syed MBJ, Aitman TJ, Newby DE, Walker NL (2020). Inherited thoracic aortic disease: New insights and translational targets. Circulation.

[3] Melvinsdottir IH, Lund SH, Agnarsson BA, Sigvaldason K, Gudbjartsson T, Geirsson A (2016). The incidence and mortality of acute thoracic aortic dissection: results from a whole nation study. Eur J Cardiothorac Surg.

[4] Fletcher AJ, Nash J, Syed MBJ, Macaskill MG, Tavares AAS, Walker N (2022). Microcalcification and thoracic aortopathy: a window into Disease Severity. Arterioscler Thromb Vasc Biol.

[5] Lanzer P, Hannan FM, Lanzer JD, Janzen J, Raggi P, Furniss D (2021). Medial arterial calcification: JACC state-of-the-art review. J Am Coll Cardiol.

[6] Irkle A, Vesey AT, Lewis DY, Skepper JN, Bird JL, Dweck MR (2015). Identifying active vascular microcalcification by (18)F-sodium fluoride positron emission tomography. Nat Commun.

[7] Tzolos E, Dweck MR (2020). (18)F-Sodium fluoride ((18)F-NaF) for imaging microcalcification activity in the Cardiovascular System. Arterioscler Thromb Vasc Biol.

[8] Syed MBJ, Fletcher AJ, Debono S, Forsythe RO, Williams MC, Dweck MR (2022). (18)F-Sodium Fluoride Positron Emission Tomography and computed tomography in Acute Aortic Syndrome. JACC Cardiovasc Imaging.

[9] Erbel R, Aboyans V, Boileau C, Bossone E, Bartolomeo RD, Eggebrecht H (2014). 2014 ESC guidelines on the diagnosis and treatment of aortic diseases: document covering acute and chronic aortic diseases of the thoracic and abdominal aorta of the adult. The Task Force for the diagnosis and treatment of aortic diseases of the European Society of Cardiology (ESC). Eur Heart J.

[10] Martinez-Möller A, Souvatzoglou M, Delso G, Bundschuh RA, Chefd’hotel C, Ziegler SI (2009). Tissue classification as a potential approach for attenuation correction in whole-body PET/MRI: evaluation with PET/CT data. J Nucl Med.

[11] Andrews JPM, MacNaught G, Moss AJ, Doris MK, Pawade T, Adamson PD (2021). Cardiovascular 18F-fluoride positron emission tomography-magnetic resonance imaging: a comparison study. J Nuclear Cardiol.

[12] Robson PM, Dweck MR, Trivieri MG, Abgral R, Karakatsanis NA, Contreras J (2017). Coronary artery PET/MR imaging: feasibility, limitations, and solutions. JACC: Cardiovasc Imaging.

[13] Michelena HI, Della Corte A, Evangelista A, Maleszewski JJ, Edwards WD, Roman MJ (2021). International consensus statement on nomenclature and classification of the congenital bicuspid aortic valve and its aortopathy, for clinical, surgical, interventional and research purposes. J Thorac Cardiovasc Surg.

[14] Nishimura RA, Otto CM, Bonow RO, Carabello BA, Erwin JP, Guyton RA (2014). 2014 AHA/ACC Guideline for the management of patients with Valvular Heart Disease: executive summary: a report of the American College of Cardiology/American Heart Association Task Force on Practice guidelines. Circulation.

[15] Fletcher AJ, Lembo M, Kwiecinski J, Syed MBJ, Nash J, Tzolos E (2021). Quantifying microcalcification activity in the thoracic aorta. J Nuclear Cardiol.

[16] Debono S, Nash J, Fletcher AJ, Syed MBJ, Semple SI, van Beek EJR (2022). Quantifying sodium [18F]fluoride uptake in abdominal aortic aneurysms. EJNMMI Res.

[17] Koo TK, Li MY (2016). A Guideline of selecting and reporting Intraclass correlation coefficients for Reliability Research. J Chiropr Med.

[18] Bland JM, Altman DG (1986). Statistical methods for assessing agreement between two methods of clinical measurement. Lancet.

[19] Chen Y, An H (2017). Attenuation correction of PET/MR Imaging. Magn Reson Imaging Clin N Am.

[20] Keereman V, Mollet P, Berker Y, Schulz V, Vandenberghe S (2013). Challenges and current methods for attenuation correction in PET/MR. Magma.

[21] Keane MG, Wiegers SE, Plappert T, Pochettino A, Bavaria JE, Sutton MG (2000). Bicuspid aortic valves are associated with aortic dilatation out of proportion to coexistent valvular lesions. Circulation.

[22] Fletcher AJ, Tew YY, Tzolos E, Joshi SS, Kaczynski J, Nash J (2022). Thoracic aortic (18)F-Sodium fluoride activity and ischemic stroke in patients with established Cardiovascular Disease. JACC Cardiovasc Imaging.

[23] Wanga S, Hibender S, Ridwan Y, van Roomen C, Vos M, van der Made I (2017). Aortic microcalcification is associated with elastin fragmentation in Marfan syndrome. J Pathol.

[24] Forsythe RO, Dweck MR, McBride OMB, Vesey AT, Semple SI, Shah ASV (2018). 18)F-Sodium fluoride uptake in Abdominal aortic aneurysms: the SoFIA(3) study. J Am Coll Cardiol.

[25] Chaibi H, Nourine R (2018). New Pseudo-CT Generation Approach from Magnetic Resonance Imaging using a local texture descriptor. J Biomed Phys Eng.

