# Supplementary material for: Thoracic aortic microcalcification activity in combined positron emission tomography and magnetic resonance imaging
Source: Eur J Nucl Med Mol Imaging. 2024 Mar 8;51(8):2260–70. doi: 10.1007/s00259-024-06670-5 (PMC11178619; doi:10.1007/s00259-024-06670-5)
Supplement: Supplementary file 1 — Supplementary Material 1 [file 259_2024_6670_MOESM1_ESM.docx]

# Supplementary Materials

# Thoracic Aortic Microcalcification Activity in Combined Positron Emission Tomography and Magnetic Resonance Imaging

Jennifer Nash^1^, Samuel Debono^1^, Beth Whittington^1^, Jakub Kaczynski^1^, Tim Clark^1^, Gillian Macnaught^1,2^, Scott Semple^1,3^, Edwin J R van Beek^1,3^, Adriana Tavares^1^, Damini Dey^4^, Michelle C Williams^1^, Piotr J Slomka^4^, David E Newby^1^, Marc R Dweck^1^, Alexander J Fletcher^5^

**Author Affiliations**

^1^ The University of Edinburgh Centre for Cardiovascular Science, University of Edinburgh, United Kingdom

^2^ Department of Medical Physics, NHS Lothian, Royal Infirmary of Edinburgh, United Kingdom

^3^ Edinburgh Imaging Facility Queens Medical Research Institute, University of Edinburgh, United Kingdom

^4^ Departments of Medicine (Division of Artificial Intelligence) and Biomedical imaging Research Institute, Cedars-Sinai Medical Centre, Los Angeles, United States

^5^School of Cardiovascular and Metabolic Health, University of Glasgow, United Kingdom

**Address for correspondence**

Dr Jennifer Nash

Clinical Research Fellow

Room SU.305, Chancellor’s Building, University of Edinburgh, 49 Little France Crescent, Edinburgh, United Kingdom, EH16 4SB

**Telephone:** +44 (0) 131 242 6515

**E-mail:** jennifer.nash@ed.ac.uk | **ORCID:** 0000-0001-7523-1127

**Supplementary Table 1.** Echocardiography aortic valve measurements for enrolled patients with bicuspid aortic valve

|  | Median [quartile interval] |
| --- | --- |
| Aortic valve velocity (m/s) |  |
| Vmax | 2.12 [1.58 – 2.54] |
| Vmean | 1.51 [1.25 – 1.80] |
| Aortic valve pressure gradient (mmHg) |  |
| Maximum pressure gradient | 18.1 [9.89 – 25.8] |
| Mean pressure gradient | 10.5 [7.08 – 14.7] |

Vmax = maximum aortic velocity, Vmean = mean aortic velocity

**Supplementary Table 2.** Levels of agreement are assessed in three different methods of measuring aortic sodium [^18^F]fluoride uptake.

|  | Mean bias | 95% limits of agreement | Intraclass correlation coefficient |
| --- | --- | --- | --- |
| **Total aortic SUV** |  |  |  |
| PET-CT and Dixon | 5.58 | -23.38 to 34.55 | 0.84 |
| PET-CT and RadialVIBE-4 | 3.29 | -32.77 to 39.35 | 0.77 |
| PET-CT and RadialVIBE-2 | 7.53 | -20.96 to 36.01 | 0.85 |
| **Aortic SUVmean** |  |  |  |
| PET-CT and Dixon | 0.01 | -0.27 to 0.29 | 0.67 |
| PET-CT and RadialVIBE-4 | -0.02 | -0.35 to 0.30 | 0.59 |
| PET-CT and RadialVIBE-2 | 0.05 | -0.25 to 0.34 | 0.62 |
| **AMA** |  |  |  |
| PET-CT and Dixon | -0.58 | -0.1 to 0.38 | 0.35 |
| PET-CT and RadialVIBE-4 | -0.02 | -0.42 to 0.37 | 0.29 |
| PET-CT and RadialVIBE-2 | -0.03 | -0.41 to 0.34 | 0.41 |

Results presented represent the mean bias and 95% limits of agreement in absolute values in each method of quantification.

AMA = aortic microcalcification activity, aortic SUVmean = aortic mean standardised uptake value per volume in cm^3^ not corrected for blood pool, total aortic SUV = aortic standardised uptake value not corrected for blood pool or volume, PET-CT = positron emission tomography – computed tomography

**Supplementary Figure 1.**

**
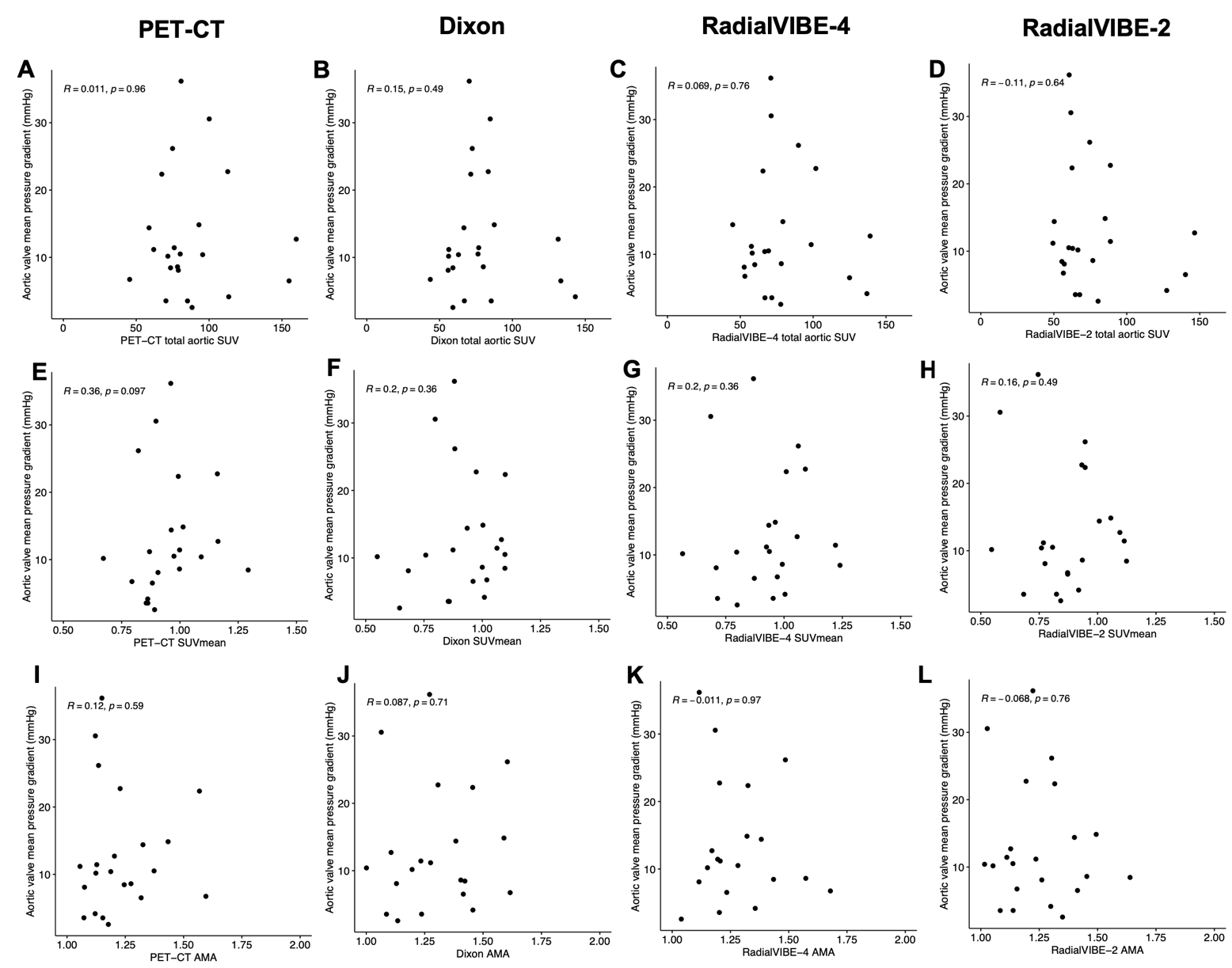
**

**Supplementary Figure 1.** Associations between aortic valve mean pressure gradient and ascending aortic ^18^F-sodium fluoride positron emission tomography uptake. A-D represent correlations between mean pressure gradient (mmHg) and total aortic standardised uptake (SUV) in positron emission tomography combined with computed tomography (PET-CT), Dixon positron emission tomography combined with magnetic resonance imaging (PET-MRI), RadialVIBE-4 PET-MRI and RadialVIBE-2 PET-MRI. E-H represent correlations between mean pressure gradient and mean standardised uptake valve (SUVmean) in each reconstruction. I-L represent correlations between mean pressure gradient and aortic microcalcification activity (AMA) in each reconstruction. Correlation coefficients (R) and p values are determined using Spearman’s rank-order correlation.

**Supplementary Figure 2**

**
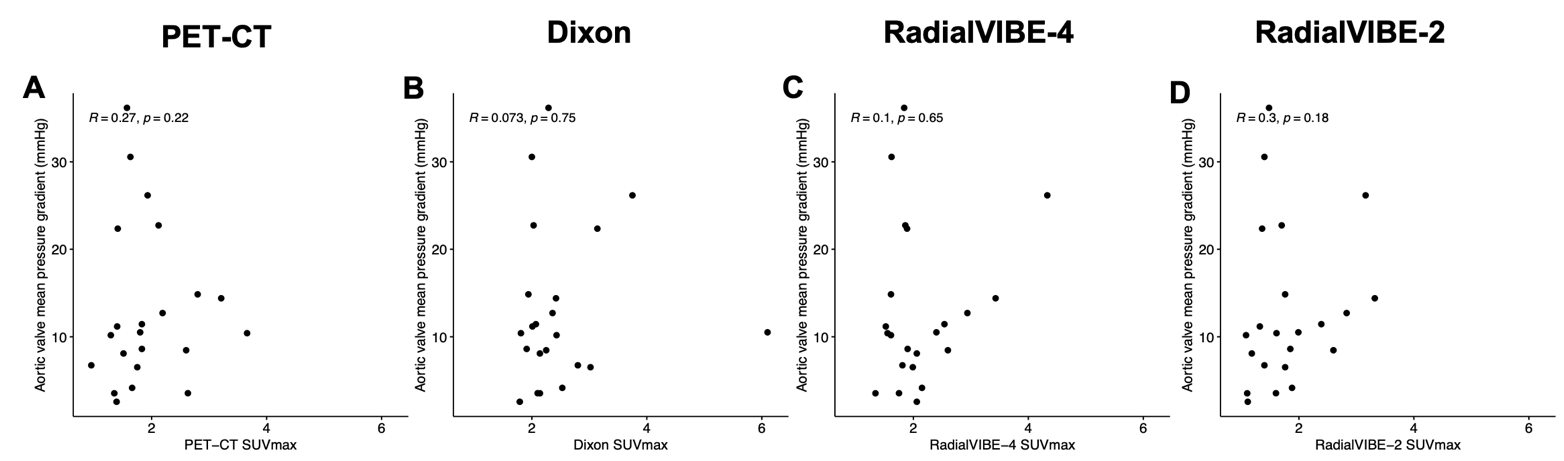
**

**Supplementary Figure 2.** Associations between aortic valve mean pressure gradient and ascending aortic sodium [^18^F]fluoride positron emission tomography maximum standardised uptake values (SUVmax). Correlations are shown between mean pressure gradient (mmHg) and SUVmax in (A) positron emission tomography combined with computed tomography (PET-CT), (B) Dixon positron emission tomography combined with magnetic resonance imaging (PET-MRI), (C) RadialVIBE-4 PET-MRI and (D) RadialVIBE-2 PET-MRI.

Correlation coefficients (R) and p values are determined using Spearman’s rank-order correlation.

**Supplementary Figure 3**

**
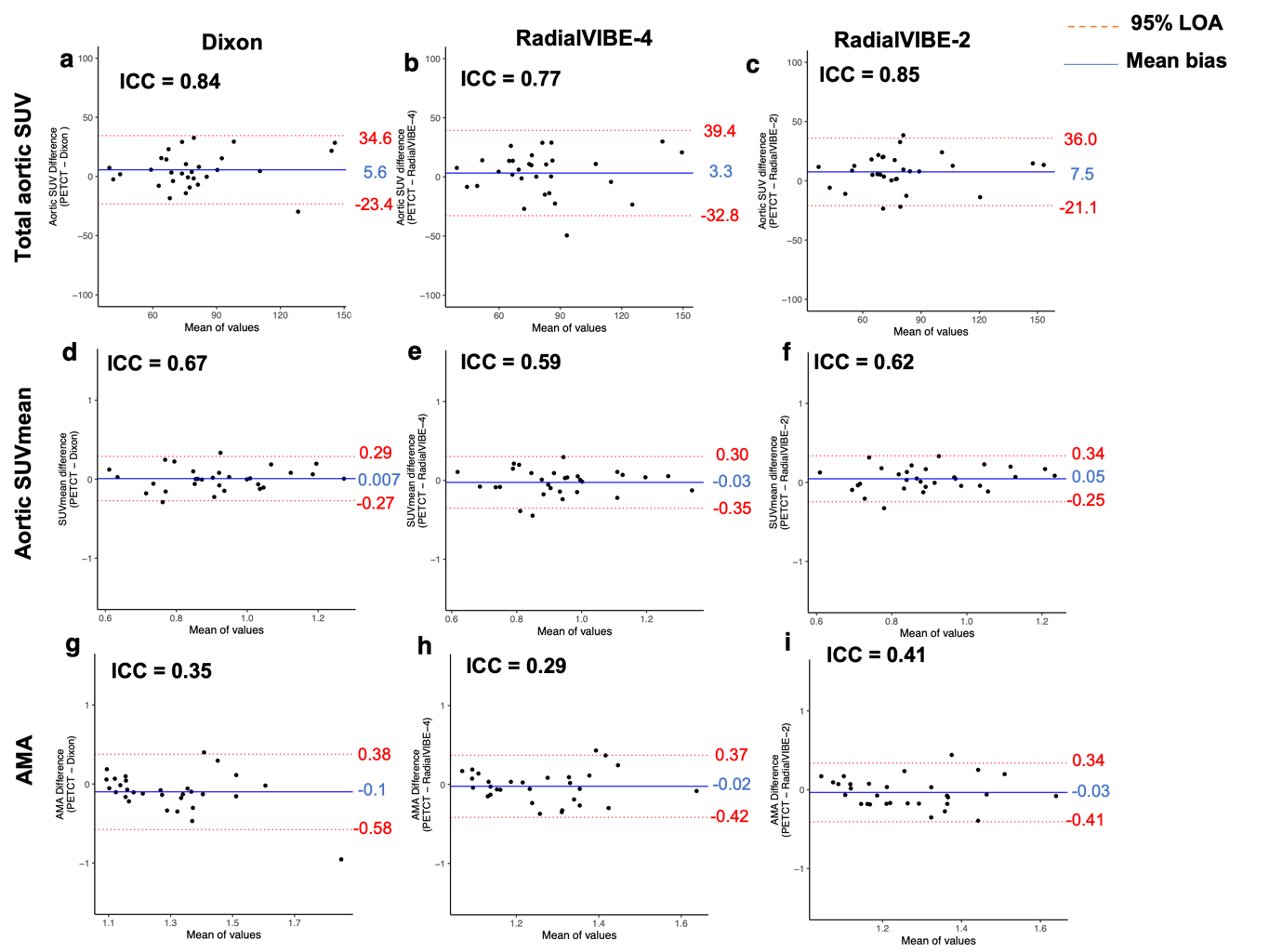
**

**Supplementary Figure 3.** Levels of agreement are assessed between positron emission tomography computed tomography (PET-CT) and positron emission tomography magnetic resonance imaging (PET-MRI) using three different methods of measuring aortic sodium [^18^F]fluoride uptake.

Bland-Altman plots demonstrate absolute difference in values with mean bias (blue line) and 95% limits of agreement (red lines) for aortic sodium [^18^F]fluoride activity. The columns represent the PET-MRI attenuation correction method compared with PET-CT. (a-c) represent total aortic standardised uptake value (SUV), (d-f) represent aortic mean standardised uptake (SUVmean), and (g-i) represent aortic microcalcification activity (AMA)

ICC = intraclass correlation coefficient, LOA = limits of agreement

**Supplementary Figure 4**

**
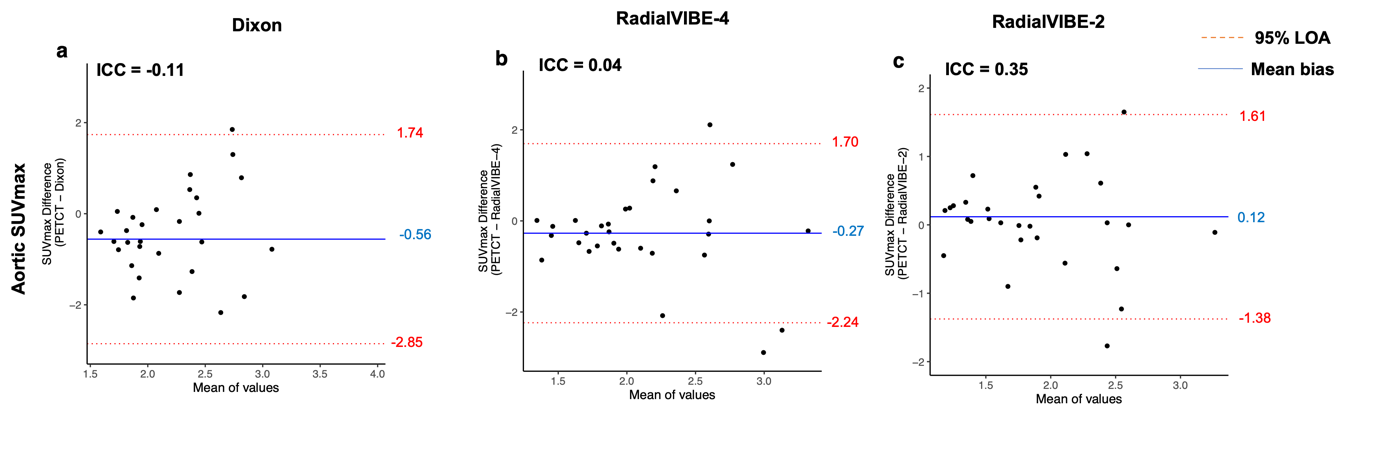
**

**Supplementary Figure 4.** Levels of agreement between positron emission tomography computed tomography (PET-CT) and positron emission tomography magnetic resonance imaging (PET-MRI) in maximum standardised uptake value (SUVmax) of aortic sodium [^18^F]fluoride uptake.

Bland-Altman plots demonstrates absolute difference in SUVmax values with mean bias (blue line) and 95% limits of agreement (red lines) for maximum aortic ^18^F-sodium fluoride activity. (a) Dixon positron PET-MRI attenuation correction method compared with PET-CT, (b) RadialVIBE-4 PET-MRI compared with PET-CT, and (c) RadialVIBE-2 PET-MRI compared with PET-CT.

ICC = intraclass correlation coefficient, LOA = limits of agreement, SUVmax = maximum standardised uptake value.
